# Effect of Trehalose Supplementation on Autophagy and Cystogenesis in a Mouse Model of Polycystic Kidney Disease

**DOI:** 10.3390/nu11010042

**Published:** 2018-12-25

**Authors:** Li-Fang Chou, Ya-Lien Cheng, Chun-Yih Hsieh, Chan-Yu Lin, Huang-Yu Yang, Yung-Chang Chen, Cheng-Chieh Hung, Ya-Chung Tian, Chih-Wei Yang, Ming-Yang Chang

**Affiliations:** Kidney Research Center, Department of Nephrology, Chang Gung Memorial Hospital, Chang Gung University College of Medicine, Taoyuan, Taiwan; d928209@adm.cgmh.org.tw (L.-F.C.); yolien0205@gmail.com (Y.-L.C.); joyyhs@gmail.com (C.-Y.H.); r5234@adm.cgmh.org.tw (C.-Y.L.); hyyang01@gmail.com (H.-Y.Y.); cyc2356@adm.cgmh.org.tw (Y.-C.C.); cchung@adm.cgmh.org.tw (C.-C.H.); dryctian@adm.cgmh.org.tw (Y.-C.T.); cwyang@ms1.hinet.net (C.-W.Y.)

**Keywords:** autophagy, polycystic kidney disease, trehalose

## Abstract

Autophagy impairment has been demonstrated in the pathogenesis of autosomal dominant polycystic kidney disease (ADPKD) and could be a new target of treatment. Trehalose is a natural, nonreducing disaccharide that has been shown to enhance autophagy. Therefore, we investigated whether trehalose treatment reduces renal cyst formation in a *Pkd1*-hypomorphic mouse model. *Pkd1* miRNA transgenic (*Pkd1* miR Tg) mice and wild-type littermates were given drinking water supplemented with 2% trehalose from postnatal day 35 to postnatal day 91. The control groups received pure water or 2% sucrose for the control of hyperosmolarity. The effect on kidney weights, cystic indices, renal function, cell proliferation, and autophagic activities was determined. We found that *Pkd1* miR Tg mice had a significantly lower renal mRNA expression of autophagy-related genes, including *atg5*, *atg12, ulk1, beclin1*, and *p62*, compared with wild-type control mice. Furthermore, immunohistochemical analysis showed that cystic lining cells had strong positive staining for the p62 protein, indicating impaired degradation of the protein by the autophagy-lysosome pathway. However, trehalose treatment did not improve reduced autophagy activities, nor did it reduce relative kidney weights, plasma blood urea nitrogen levels, or cystatin C levels in *Pkd1* miR Tg mice. Histomorphological analysis revealed no significant differences in the renal cyst index, fibrosis score, or proliferative score among trehalose-, sucrose-, and water-treated groups. Our results demonstrate that adding trehalose to drinking water does not modulate autophagy activities and renal cystogenesis in *Pkd1*-deficient mice, suggesting that an oral supplement of trehalose may not affect the progression of ADPKD.

## 1. Introduction

Autosomal dominant polycystic kidney disease (ADPKD) is characterized by progressive cyst formation and loss of renal function with age, accounting for up to 10% of patients entering treatment for end-stage renal disease [1]. The disease is caused by mutations in *PKD1* or *PKD2*, which encode the integral membrane proteins polycystin-1 and polycystin-2, respectively [2]. Insufficiency of polycystin-1 or polycystin-2 causes cellular dysfunction, such as fluid secretion, proliferation, mechanosensation, and extracellular matrix defects, and leads to the development of multiple fluid-filled cysts and the destruction of the renal parenchyma [3,4]. Advances made toward understanding the molecular and cellular mechanisms of ADPKD have led to new treatments in preclinical and clinical studies [5,6,7,8]. The Food and Drug Administration has approved the use of tolvaptan, a selective vasopressin V2 receptor (V2R) antagonist that may reduce renal cyclic adenosine monophosphate (cAMP) levels and inhibit cyst growth, for the treatment of ADPKD [9]. Nonetheless, the development of novel treatments that can act synergistically or have fewer side effects may improve treatment outcomes.

Autophagy is a lysosomal catabolic process that maintains cellular homeostasis by degrading damaged intracellular organelles and cytoplasmic components; such degradation is critical for cell survival under unfavorable growth conditions, such as energy deprivation [10]. Autophagy activities of cells are tightly regulated by both the mammalian target of rapamycin (mTOR)-dependent and -independent mechanisms [11,12,13]. Recent evidence has indicated that autophagy suppression may contribute to the pathogenesis of ADPKD, whereas chemical enhancers of autophagy may have therapeutic benefits [13,14,15]. A seminal study showed that autophagy activation through various autophagy enhancers may reduce cystogenesis in a zebrafish model of *PKD1* [16], suggesting that autophagy activation may be a new treatment strategy for ADPKD.

Trehalose is a nonreducing disaccharide consisting of two glucose molecules linked by an α, α-1,1-glucosidic bond; it is found in plants, insects, microorganisms, and invertebrates, but not in mammals [17,18,19]. Trehalose has stable physical properties and can protect the integrity of cells against various injuries, such as cold, heat, dehydration, oxidation, and hypoxia, by reducing protein denaturation through protein–trehalose interactions [20]. Trehalose has been widely used in the preservation of food, but has also been used to treat medical diseases due to its ability to enhance autophagy. In healthy middle-aged and older adults, oral trehalose supplements (100 g/day) improved microvascular function by increasing endothelial nitric oxide [21]. The protective effects of trehalose have largely been demonstrated in neurodegenerative disorders, including Alzheimer’s disease, Parkinson’s disease, and Huntington’s disease [22,23,24]. Trehalose enhanced autophagy and reduced abnormal protein aggregation in the brain tissue of mice with Huntington’s disease [24,25]. Furthermore, trehalose exerted cytoprotective effects in puromycin aminonucleoside-treated podocytes and cadmium-treated proximal tubular cell lines by inducing autophagy [26,27]. However, the role of trehalose in protecting against the progression of ADPKD remains unclear.

In this study, we tested the hypothesis that trehalose supplementation decreases renal cyst formation and progression in a mouse model of ADPKD. In addition, we determined whether autophagy is abnormally regulated during the progression of polycystic kidney disease.

## 2. Materials and Methods

### 2.1. Animals

*Pkd1* miRNA transgenic (*Pkd1* miR Tg) mice with a C57BL/6 background and wild-type littermates were provided by Si-Tse Jiang (National Rodent Model Resource Center, Taiwan). The generation of *Pkd1* miR Tg mice has been reported previously in detail [28]. In brief, the *Pkd1* miR Tg mice were generated with the expression of miRNA hairpins specific to the *Pkd1* transcript. These mice have about a 70% reduction of *Pkd1* expression and develop severe renal cysts gradually, mimicking the disease progression in human ADPKD. All of the mice were housed in the Chang Gung Memorial Hospital Animal Center (Taoyuan, Taiwan) under climate-controlled conditions with a 12-h light–dark cycle. The mice had free access to standard laboratory chow. The animal experimental protocols were approved by the Animal Care and Use Committee of Chang Gung Memorial Hospital in accordance with the National Institute of Health Guide for the Care and Use of Laboratory Animals.

### 2.2. Experimental Design

**Figure 1:**
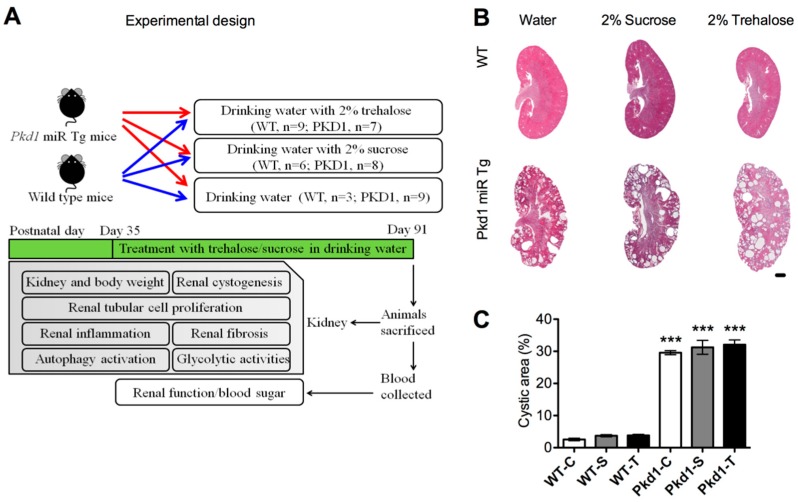
**Effect of trehalose treatment on renal cyst formation in *Pkd1* miRNA transgenic mice.** (**A**) Schematic diagram summarizes the experimental design. (**B**) Representative kidney sections stained with hematoxylin and eosin and (**C**) renal cystic indices of three-month-old *Pkd1* miR Tg mice of the different treatment groups (*n* = 3, 6, 9, 9, 8, and 7 per group, respectively). Data represent mean ± standard error of the mean. *** *p* < 0.001 compared with the wild-type control group. WT, wild-type; PKD, polycystic kidney disease; Tg, transgenic; C, control; S, sucrose; T, trehalose. Scale bar, 1 mm.

Between five (day 35) and 13 weeks (day 91) of age, the mice were given drinking water supplemented with or without 2% trehalose (w/v) (Figure 1A). An additional control group was given drinking water supplemented with 2% (w/v) sucrose to control the effect of hyperosmolarity. The dosages of trehalose and sucrose were selected according to previous studies on various mouse models [25,29,30]. Trehalose and sucrose were purchased from Sigma-Aldrich (St. Louis, MO, USA).

The mice were anesthetized and sacrificed on day 91. The kidneys were harvested from all sacrificed animals. The ratio of the terminal combined weight of the two kidneys to the body weight (fraction kidney weight) was compared among the treatment groups. For assessing the degree of cell proliferation, all of the mice received an intraperitoneal injection of 5-bromo-2′-deoxyuridine (BrdU, 0.3 mg/kg) (Sigma-Aldrich, St. Louis, MO, USA) four hours before sacrifice. Blood samples were collected using a heparinized syringe through cardiac puncture under anesthesia. Non-fasting glucose levels were immediately determined using a portable glucometer. Plasma blood urea nitrogen (BUN) levels were determined using a mouse-specific enzyme-linked immunosorbent assay (ELISA) kit (Bioassay Systems, Hayward, CA, USA), according to the manufacturer’s instructions. We also measured plasma cystatin C levels for renal function estimation using a mouse/rat cystatin C Quantikine ELISA kit (R&D systems, Minneapolis, MN, USA) [31].

### 2.3. Histomorphometric Analysis

Transverse kidney sections (4-μm thick) were stained with hematoxylin and eosin and Masson’s trichrome stain [32]. The images were analyzed using MetaMorph software (Universal Imaging, West Chester, PA, USA). The cystic index was calculated as a percentage by dividing the area of the cystic lumen by the area of the renal tissue and lumen [33]. The fibrotic area percentage was calculated using five consecutively selected fields of the renal cortex and medulla at 100× magnification in the sections stained with Masson’s trichrome stain. All analyses were performed on coded slides in a single-blinded manner.

### 2.4. Immunohistochemistry

Immunohistochemical staining was performed on paraffin-embedded kidney sections after being deparaffinized in xylene and hydrated in a graded series of alcohol. These steps were followed by antigen retrieval using 2 N HCl or citrate buffer (pH 6.0). The sections were blocked with the blocking reagents (Dako antibody diluent, Cat. No. S3022; Dako Agilent Technologies, Santa Clara, CA, USA). BrdU-positive cells on paraffin sections were detected by incubating the sections in a rabbit monoclonal anti-BrdU antibody (1:1000, ab6326, Abcam, Cambridge, UK) after antigen retrieval using 2 N HCl [34]. The proliferative index was determined by counting the number of BrdU-positive epithelial cells at 200× magnification in each renal section in sequentially selected fields. To label the autophagy marker p62, a rabbit polyclonal antibody p62 (SQSTM1) (1:1000, PM045; MBL, Nagoya, Japan) was used after antigen retrieval using citrate buffer (pH 6.0). A rabbit-on-rodent horseradish peroxidase (HRP) polymer (RMR622H; Biocare, Concord, CA, USA) and 3,3′-diaminobenzidine chromogen (K3468; Dako, Glostrup, Denmark) were used to visualize staining. The slides were counterstained with hematoxylin (Muto Pure Chemicals, Tokyo, Japan).

Immunofluorescence staining was performed on paraffin-embedded renal tissue sections after being dewaxed and rehydrated. Heat-induced antigen retrieval was performed in citrate buffer (pH 6.0). Samples were incubated in blocking buffer (Dako antibody diluent, Cat. No. S3022; Dako Agilent Technologies, Santa Clara, CA, USA), followed by incubation with the primary antibody for p62 (1:1000, PM045; MBL, Nagoya, Japan). Alexa Fluor 594 donkey anti-rabbit IgG (Invitrogen, Molecular Probes, Paisley, UK) was used to detect the primary antibody and the nuclei are counter stained with 4,6-diamidino-2-phenylindole (DAPI). Slides were mounted using Fluorsave aqueous mounting medium (Calbiochem, La Jolla, CA, USA) and observed under an Olympus BX50 microscope (Olympus, Hamburg, Germany).

### 2.5. Real-Time Polymerase Chain Reaction

Snap-frozen mouse kidneys were ground in liquid nitrogen. An RNA-Bee isolation kit (Tel-Test, Friendswood, TX, USA) was used for total RNA extraction. cDNA was obtained from the total RNA by using a first-strand cDNA synthesis kit (Transcriptor cDNA Synthesis kit, Cat. No. 04897030001; Roche Diagnostics, Mannheim, Germany). Real-time PCR was performed using an ABI ViiA7 sequence detection system (Applied Biosystems, Foster City, CA, USA) coupled with a SYBR Green or TaqMan assay (Supplementary Table S1), following the manufacturer’s instructions. For each sample, the reactions were performed in duplicate. The relative mRNA expression levels were calculated using the 2-ddCt method.

### 2.6. Western Blotting

Total proteins were extracted from snap-frozen kidney specimens following standard protocols [33]. Briefly, tissue samples were homogenized in ice-cold lysis buffer and sonicated with a Vibra Cell VCX-500 sonicator (Sonics & Materials, Newtown, CT, USA). Cell debris was removed by centrifugation at 14,000 rpm for 15 min at 4 °C. Concentrations of the protein supernatants were determined with a Bradford protein assay (Bio-Rad, Hercules, CA, USA) and equal amounts of total protein were separated by sodium dodecyl sulfate–polyacrylamide gel electrophoresis. After separating, the resulting proteins were then transferred onto a polyvinylidene fluoride membrane (Millipore, Bedford, MA, USA). After blocking in 5% fat-free milk, the membrane was incubated individually with the primary antibodies including rabbit polyclonal anti-p62 (1:1000, PM045; MBL, Nagoya, Japan), rabbit anti-LC3 (1:500, Novus Biologicals, Littleton, CO, USA), and mouse anti-β-actin (1:10,000, AC-15; Abcam, Cambridge, UK) at 4 °C overnight, and further incubated with horseradish peroxidase-conjugated secondary antibodies. Protein signals were examined using Amersham ECL Prime Western Blotting Detection Reagent (Cat. No. RPN2232; GE Healthcare, Buckinghamshire, UK), according to the manufacturer’s instructions.

### 2.7. Statistical Analysis

Values are expressed as the mean ± standard error of the mean. Between-group comparisons were performed using one-way analysis of variance, followed by the Dunnett post hoc test. *p* values < 0.05 were considered statistically significant. All analyses were performed using GraphPad Prism 5.1 (GraphPad, La Jolla, CA, USA).

## 3. Results

### 3.1. Effect of Trehalose Treatment on Kidney and Body Weight

In this study, we utilized *Pkd1* miR Tg mice, an orthologous mouse model mimicking the natural progression of ADPKD, to test the potential therapeutic efficacy of trehalose [28]. Five-week-old male *Pkd1* miR Tg mice and wild-type littermates (*n* = 3–9 in each group) were fed drinking water supplemented with 2% trehalose for eight weeks. The control groups were fed pure drinking water or drinking water supplemented with 2% sucrose. We first examined the fraction of kidney weight as a parameter of disease severity. As shown in Table 1, *Pkd1* miR Tg mice had a significantly higher kidney weight-to-body weight ratio than wild-type mice (*p* < 0.001). However, the oral administration of trehalose did not decrease the fraction of kidney weight of *Pkd1* miR Tg mice compared with those in the mice fed drinking water supplemented with 2% sucrose or pure drinking water. The body weights of *Pkd1* miR Tg mice fed drinking water supplemented with trehalose, drinking water supplemented with sucrose, or pure drinking water were not significantly different. However, *Pkd1* miR Tg mice in the sucrose group had significantly lower body weights than wild-type control mice (*p* < 0.05).

### 3.2. Effect of Trehalose Treatment on Renal Function and Blood Sugar

We examined the effect of trehalose treatment on renal function and blood sugar (Table 1) in the mice. *Pkd1* miR Tg mice had a 1.8-fold increase in plasma BUN levels compared with wild-type mice (42.1 ± 1.8 vs. 23.2 ± 1.1 mg/dL, respectively, *p* < 0.001). However, the addition of 2% trehalose to drinking water did not significantly affect the BUN levels of either wild-type or *Pkd1* miR Tg mice. Similarly, plasma cystatin C levels were not significantly different among the three treatment groups. The mice treated with trehalose or sucrose tended to have higher blood glucose levels, although the differences were not statistically significant (Table 1).

### 3.3. Effect of Trehalose on Renal Cystogenesis

**Figure 2:**
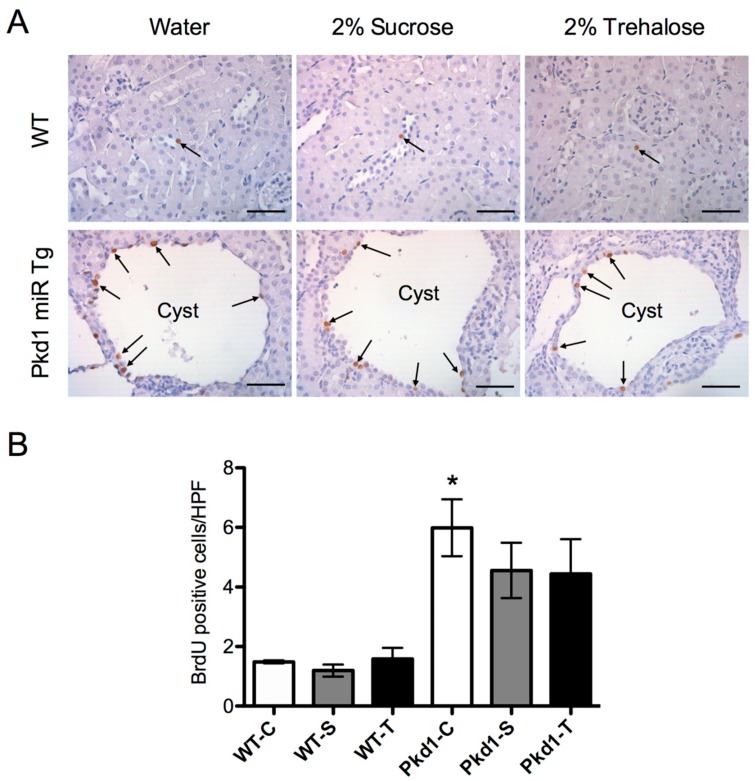
**Renal tubular cell proliferation in trehalose-treated*Pkd1*miRNA transgenic mice versus controls.** (**A**) Representative images of BrdU staining (arrows) of renal tubules and cysts in three-month-old wild-type and *Pkd1* miR Tg mouse kidney sections following trehalose or control treatment. (**B**) Quantitative analysis of BrdU staining (*n* = 3, 6, 9, 9, 8, and 7 per group, respectively). * *p* < 0.05 compared with the wild-type control group. WT, wild-type; PKD, polycystic kidney disease; Tg, transgenic; C, control; S, sucrose; T, trehalose; HPF, high-power field. Scale bar, 50 µm.

To investigate whether trehalose treatment prevents cyst growth in *Pkd1* miR Tg mice, we determined the percentage of cystic area in both kidneys through histomorphological analysis. As demonstrated in Figure 1B,C, cystic indices were not significantly different among the three treatment groups. We also examined the proliferation of renal epithelial cells by counting the number of BrdU-positive cells. The number of BrdU-positive tubular epithelial cells (cystic and noncystic) significantly increased by more than five-fold in *Pkd1* miR Tg mice compared with wild-type control mice (Figure 2). However, the differences were not significantly influenced by the administration of trehalose. These results demonstrated that an oral supplement of trehalose did not prevent cyst growth in polycystic kidney disease.

### 3.4. Effect of Trehalose on Renal Fibrosis

**Figure 3:**
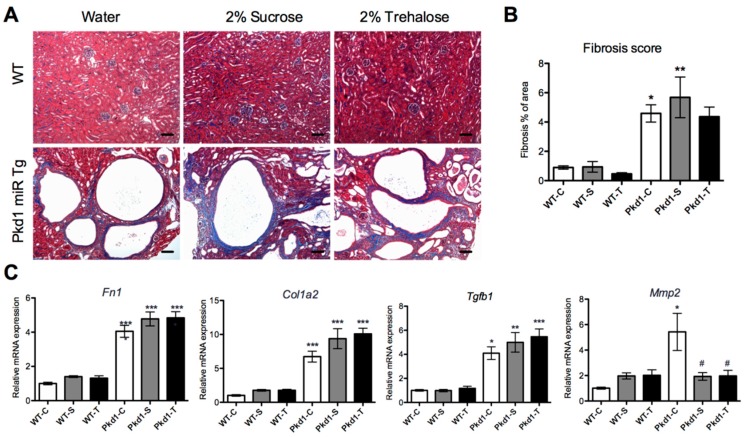
**Effect oftrehalose treatment on renal fibrosis.** (**A**) Representative images and (**B**) quantitative analysis of Masson’s trichrome staining in the renal sections of wild-type and *Pkd1* miRTg mice in the different experimental groups (*n* = 3, 6, 9, 9, 8, and 7 per group, respectively). Original magnification, 200×. (**C**) Relative renal mRNA amounts of *Fn1, Col1a2*, *Tgfb1,* and *Mmp2.* * *p* < 0.05, **p < 0.01, *** *p* < 0.001 compared with the wild-type control group. ^#^ *p* < 0.05 compared with *Pkd1* control group. WT, wild-type; PKD, polycystic kidney disease; Tg, transgenic; C, control; S, sucrose; T, trehalose. Scale bar, 50 µm.

**Figure 4:**
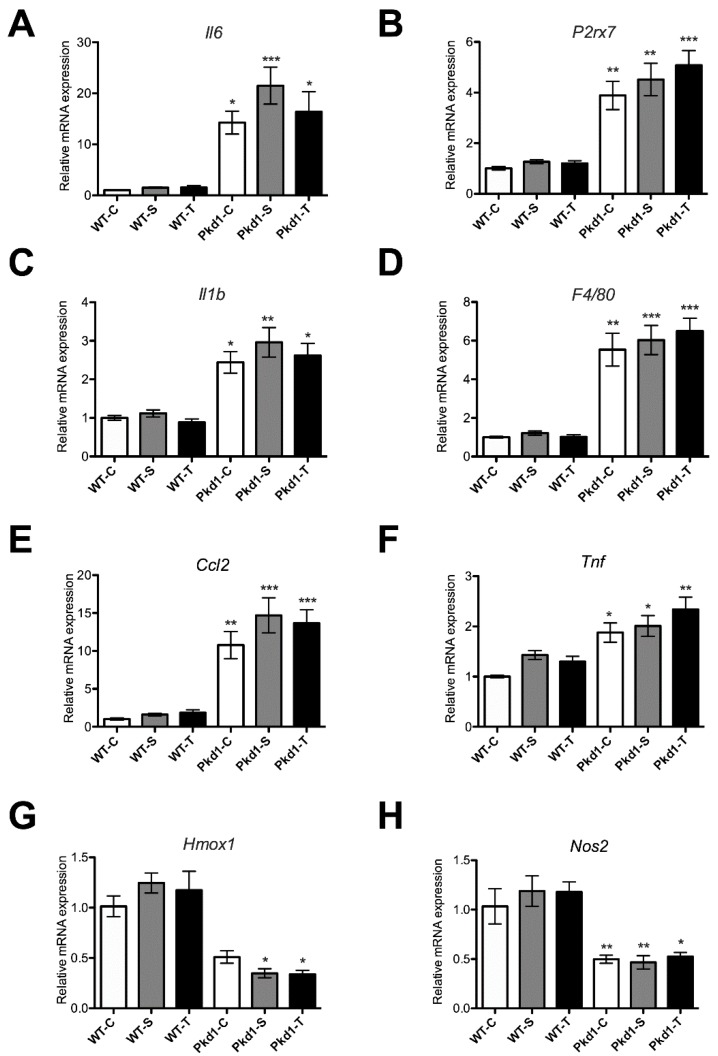
**Trehalose treatment did not affect renal inflammation in *Pkd1* miRNA transgenic mice.** The renal mRNA expression levels of (**A**) *Il6*, (**B**) *P2rx7*, (**C**) *Il1b*, (**D**) *F4/80*, (**E**) *Ccl2*, (**F**) *Tnf,* (**G**) *Hmox1,* and (**H**) *Nos2* in the different experimental groups of mice (n = 3, 6, 9, 9, 8, and 7 per group, respectively). * *p* < 0.05, ** *p* < 0.01, *** *p* < 0.001 compared with the wild-type control group. WT, wild-type; PKD, polycystic kidney disease; C, control; S, sucrose; T, trehalose.

Renal fibrosis and extracellular matrix abnormalities are prominent features of ADPKD progression [35]. As shown in Figure 3, the degree of renal fibrosis, as determined by the histomorphological analysis of Masson’s trichrome stain, significantly increased by five-fold in *Pkd1* miR Tg mice compared with wild-type control mice (*p* < 0.05). Furthermore, the mRNA expression of the renal fibrosis-related genes *Col1a2*, *Fn1,* and *Tgfb1* were all significantly increased in *Pkd1* miR Tg mice (*p* < 0.05) compared with wild-type mice. The administration of trehalose did not significantly decrease these changes compared with controls. We found a significantly lower amount of *Mmp2* mRNA in *Pkd1* miR Tg mice treated with trehalose compared with the water control group (*p* < 0.05), and a similar reduction was also seen in the sucrose group. The transcription activation of the inflammatory-related genes *Il1b*, *Il6*, *Ccl2, Tnf, F4/80,* and *P2rx7* was significantly increased (*p* < 0.05), and the expression of the anti-inflammatory genes *HO-1* and *iNOS* was significantly reduced (*p* < 0.05), in *Pkd1* miR Tg mice compared with wild-type mice. However, these changes were not affected by the administration of trehalose (Figure 4).

### 3.5. Effect of Trehalose on Autophagy Activation

**Figure 5:**
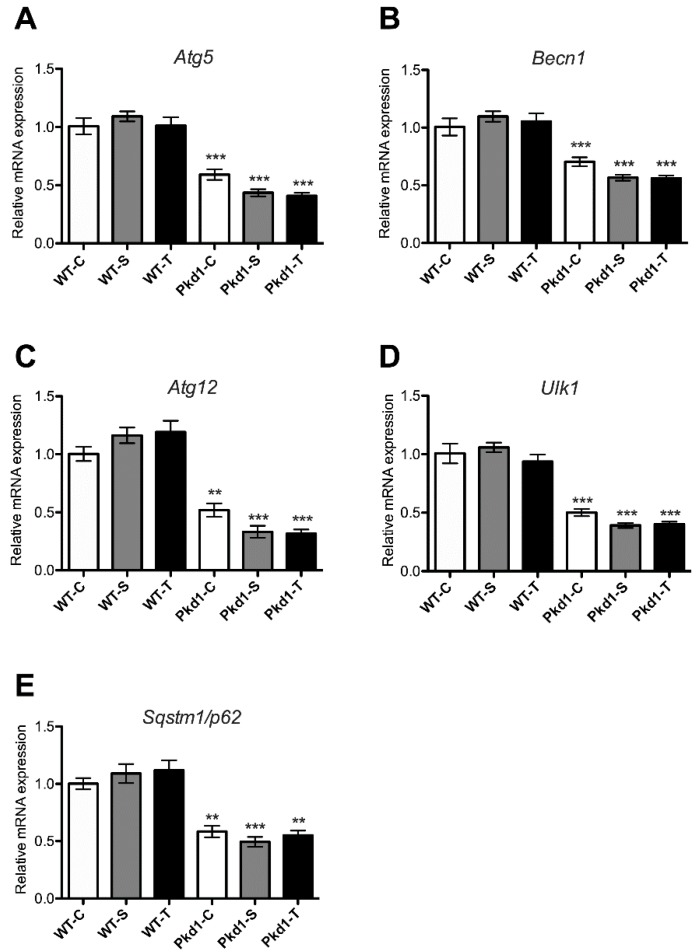
**Effect of trehalose on the expression of autophagy-related genes in the kidneys of *Pkd1* miRNA transgenic mice.** The relative renal mRNA amounts of (**A**) *Atg5,* (**B**) *Becn1,* (**C**) *Atg12,* (**D**) *Ulk1, and (E) Sqstm1/p62* in the mice in the different experimental groups compared with wild-type mice (*n* = 3, 6, 9, 9, 8, and 7 per group, respectively). ** *p* < 0.01, *** *p* < 0.001 compared with the wild-type control group. WT, wild-type; PKD, polycystic kidney disease; C, control; S, sucrose; T, trehalose.

To determine whether trehalose increases autophagy activities in *Pkd1* miR Tg mice [36], we assessed the effect of trehalose treatment on the transcription activation of *Atg5*, *Atg12, Ulk1, Becn1,* and *p62,* which encode essential proteins involved in autophagosome formation. The renal mRNA expression of these autophagy-related genes was significantly reduced in *Pkd1* miR Tg mice compared with wild-type mice (Figure 5), indicating a role of autophagy impairment in the pathogenesis of ADPKD. However, trehalose administration had no detectable effect on the mRNA expression of these autophagy-associated genes.

**Figure 6:**
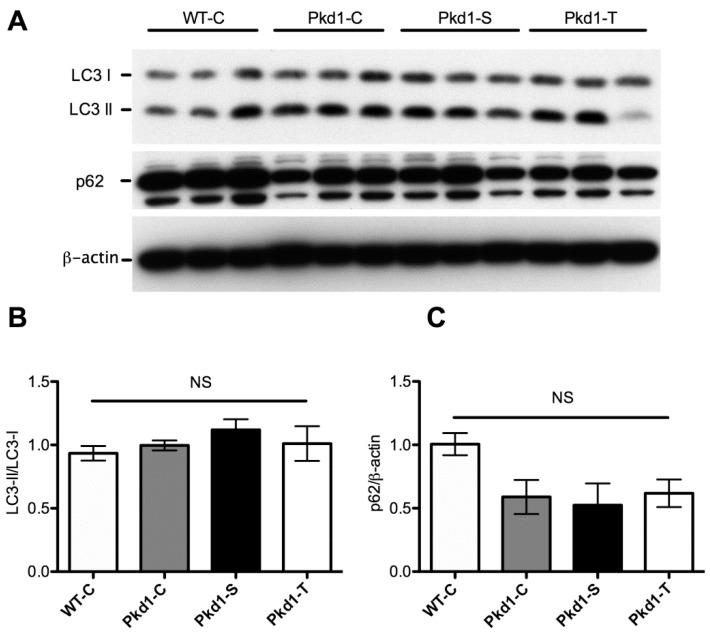
**Trehalose treatment did not enhance the renal expression of autophagy markers.** (**A**) Immunoblots of three-month-old wild-type and *Pkd1* miRNA transgenic mice in the different treatment groups are represented. The kidney lysates were immunoblotted for LC3, p62, and β-actin (for loading control). (**B**) The densitometric results of LC3II were compared with those of LC3I. (**C**) The denstiometric results of p62 were normalized to β-actin. Graphs represent mean ± standard error of the mean (*n* = 3 for wild-type mice and *n* = 6 for *Pkd1* mice per treatment group). WT, wild-type; PKD, polycystic kidney disease; C, control; S, sucrose; T, trehalose; NS, non-significant.

**Figure 7:**
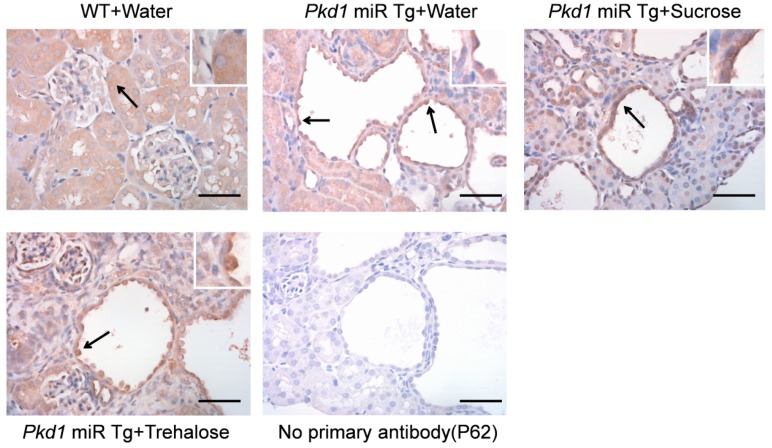
**Increased expression of p62 protein in cyst-lining epithelial cells in *Pkd1* miRNA transgenic mice.** Both wild-type and *Pkd1* miR Tg mice exhibited weak cytoplasmic p62 staining in noncystic tubular epithelial cells. Strong p62 staining (arrows) of the cystic epithelial cells in *Pkd1* miR Tg mice was present, indicating reduced autophagic degradation of the p62 protein. No significant differences in p62 staining were found among the different treatment groups. The negative control sections, without primary antibody, showed no staining. The right insets show higher magnification of the areas indicated by arrows. Representative images are shown (*n* = 3 per group). Scale bar, 50 µm.

Although the mRNA expression of these autophagy-related genes was reduced in *Pkd1* miR Tg mice, Western blot analysis of whole-kidney lysates revealed no significant difference in the expression of the microtubule-associated protein light chain 3 (LC3) II/LC3I or p62 protein between wild-type and *Pkd1* miR Tg mice (Figure 6 and Supplementary Figure S1). These findings suggest a possible defect in autophagolysosomes, which degrade these autophagy-related proteins. Immunostaining of the p62 protein, an autophagy cargo receptor protein degraded by autophagosomes [37], revealed stronger staining for cyst-lining epithelial cells than for noncystic tubular cells (Figure 7 and Supplementary Figure S2). This result indicates reduced autophagy-mediated degradation of the p62 protein in the cystic epithelium [38]. This finding is consistent with a report that demonstrated insufficient autophagic flux and inadequate autophagosome formation in a zebrafish *pkd1a* mutant and *Pkd1* mutant kidney cells [16]. However, the pattern of immunostaining for the p62 protein was not different between *Pkd1* miR Tg mice receiving drinking water supplemented with trehalose, drinking water supplemented with sucrose, or pure drinking water.

### 3.6. Effect of Trehalose on Glycolysis

**Figure 8:**
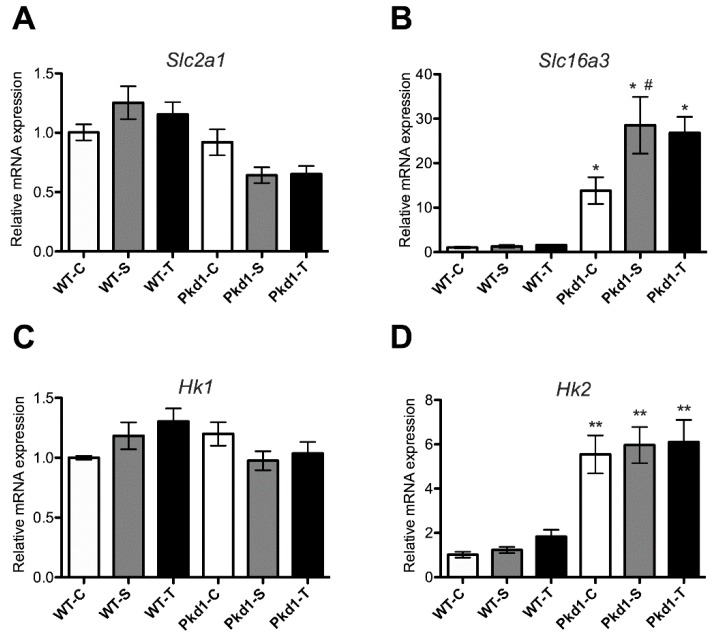
Effect of trehalose on the expression of glycolysis-related genes in the kidneys of the *Pkd1* miRNA transgenic mice. The relative renal mRNA amounts of (**A**) *Slc2a1,* (**B**) *Slc16a3,* (**C**) *Hk1,* and (**D**) *Hk2* in the mice in the different experimental groups compared with wild-type mice are presented (*n* = 3, 6, 9, 9, 8, and 7 per group, respectively). * *p* < 0.05, ** *p* < 0.01 compared with the wild-type control group. ^#^ *p* < 0.05 compared with the *Pkd1* control group. WT, wild-type; PKD, polycystic kidney disease; C, control; S, sucrose; T, trehalose.

Finally, we examined the effect of trehalose ingestion on the mRNA expression of glycolysis-related genes. Trehalose metabolism may restrict sugar influx into glycolysis by inhibiting hexokinase activities [19]. Compared with wild-type mice, *Pkd1* miR Tg mice had significantly higher levels of *Slc16a3* and *Hk2* mRNA expression (Figure 8), indicating an increase in glycolytic activities in polycystic kidneys. The increase in *Slc16a3* mRNA expression was even higher in sucrose-treated *Pkd1* miR Tg than in untreated *Pkd1* miR Tg mice (*p* < 0.05). However, trehalose administration did not inhibit the expression of these glycolytic-related genes. These results show that the oral administration of trehalose cannot rescue increased glycolytic activities in ADPKD.

## 4. Discussion

Autophagy activation has been proposed as a novel treatment for ADPKD [16]. Trehalose is a natural, nontoxic disaccharide sugar that has been shown to enhance autophagy activities in animal models of neurological disorders [30]. Therefore, in the present study, we determined whether trehalose treatment is beneficial to *Pkd1* miR Tg mice, which represent a chronic progression model of ADPKD. Despite hypothesizing that trehalose might restore autophagy function in ADPKD, we found that the oral administration of trehalose did not reduce the severity of renal cystogenesis or significantly affect autophagy activation.

A possible explanation for the lack of efficacy may be the presence of trehalase in the mouse gut and kidney, which would partially degrade trehalose into two glucose molecules [19]. However, the efficacy of orally administered trehalose has been well-documented in neurodegenerative disorders. Trehalose (2%) added to the drinking water was directly bound and inhibited polyglutamine aggregates in a mouse model of Huntington’s disease, and the presence of trehalose in the homogenates of brain and liver tissue has been confirmed, indicating the effectiveness of orally administered trehalose [25]. The long-term administration of 1% trehalose through drinking water over two and a half months reverted the death of dopamine neurons and induced autophagy in a mouse model of parkinsonism [39]. In a mouse model of Lewy body disease, the oral intake of 2% trehalose for one week was associated with increased autophagy in the brain [40]. In contrast to these studies, in the present study, we found that the oral administration of 2% trehalose for eight weeks did not enhance autophagy activities in normal or cystic mouse kidneys. These results suggest that the long-term administration of low doses of trehalose may not modulate the progression of ADPKD. We could not exclude that the administration of trehalose at higher concentrations or through a different route would be effective. For example, the intrathecal administration of trehalose has been found to be neuroprotective, and the underlying mechanism involves the reduction of MMP-2 and MMP-9 expression after spinal cord injury in mice [41]. Moreover, the combination of trehalose with other autophagy enhancers may have synergistic effects, warranting additional studies.

Autophagy impairment has been proposed to play a crucial role in the pathogenesis of ADPKD by modulating cell proliferation [42]. A defect in autophagy may predispose cyst lining cells to apoptosis and promote cyst formation in ADPKD [43]. However, the degree of autophagy impairment in different stages of cyst development, such as early versus late, could be different [13]. In our study, on the basis of the reduced renal mRNA expression of autophagy-related genes and increased expression of the p62 protein in cystic lining epithelial cells, our results confirmed the impairment of autophagy in *Pkd1*-deficient mice and supported a specific role of autophagy in the pathogenesis of ADPKD [44]. Whether the autophagy-related pathways are primary events for cyst initiation or secondary to cyst progression needs to be further investigated [45].

The pathophysiology of ADPKD has been partially attributed to metabolic derangements [46]. Polycystic kidney disease cells displayed a proproliferative glycolytic phenotype with increased lactate formation and extracellular acidification [47]. High glucose concentrations may promote cyst growth through calcium-activated chloride secretion [48], whereas glycolytic inhibitors, such as 2-deoxyglucose (2DG), have protective effects against cyst progression [49]. Trehalose ingestion may also inhibit the absorption of glucose in the gut and exert some anti-glycolysis effects [19]. However, we did not observe any changes in the expression of glycolytic enzymes in the mice treated with trehalose. Our data did not support that trehalose could be a glycolytic inhibitor.

In the current study, the mice fed drinking water supplemented with sucrose had a slightly more severe cystic phenotype than untreated mice, supporting that food restriction or calorie restriction exerts protective effects, and sugar consumption worsens ADPKD [31]. On the other hand, water intake may suppress cyst fluid secretion through the inhibition of arginine vasopressin (AVP)-dependent cAMP synthesis [50]. Although we did not measure the amount of water consumption, the mice in the sucrose group may have drunk more water than the mice fed drinking water supplemented with less sweet trehalose, which could have interfered with the results [50]. Trehalose may have also been metabolized into glucose in the mouse gut or kidney [19], which may interfere with its potential protective effects.

A limitation of the current study is that we only used one model and cannot exclude the potential effectiveness of trehalose in other models. Because the study model is a chronic progression model that resembles adults with ADPKD, we could not assess the effect of trehalose on the initiation of renal cysts and autophagy in the early stages of cystogenesis. Moreover, a higher concentration of trehalose or its combination with other autophagy enhancers should be investigated in additional studies [51,52].

In conclusion, the oral administration of trehalose in *Pkd1* miR Tg mice did not ameliorate the progression of polycystic kidney disease. The lack of beneficial effects could be due to the absence of significant autophagy impairment in the current model or the method of drug administration. These results suggest that oral trehalose supplement has no beneficial effects on preventing renal cyst formation and disease progression in ADPKD. Our data support that impaired autophagy activities are still present in the late stage of cystogenesis and might have a role in the pathogenesis of renal cysts. More potent autophagy enhancers or in combination with other targeted drugs should be investigated in additional studies.

## Figures and Tables

**Table 1:** Effect of trehalose on the metabolic and biochemical parameters of *Pkd1* miRNA transgenic mice.

Parameters	WT/C (*n* = 3)	WT/S (*n* = 6)	WT/T (*n* = 9)	PKD1/C (*n* = 9)	PKD1/S (*n* = 8)	PKD1/T (*n* = 7)
Body weight (g)	29.4 ± 1.0	28.4 ± 0.4	27.2 ± 0.3	26.7 ± 1.0	24.6 ± 0.6 *	26.1 ± 0.6
Kidney weight (g)	0.343 ± 0.025	0.319 ± 0.008	0.316 ± 0.001	0.592 ± 0.019 *	0.569 ± 0.046 *	0.584 ± 0.041 *
Kidney-to-body weight Ratio (%)	1.16 ± 0.04	1.12 ± 0.04	1.16 ± 0.03	2.23 ± 0.07 *	2.30 ± 0.17 *	2.23 ± 0.13 *
Plasma BUN (mg/dL)	23.2 ± 1.1	25.2 ± 1.0	27.9 ± 1.1	42.1 ± 1.8 *	45.9 ± 2.7 *	45.0 ± 3.5 *
Plasma cystatin C (ng/mL)	456.4 ± 11.1	454.2 ± 48.1	510.0 ± 26.8	600.7 ± 39.8	686.9 ± 47.5 *	645.1 ± 35.5
Blood sugar(mg/dL)	155.0 ± 9.6	213.8 ± 16.7	216.3 ± 12.4	196.8 ± 14.9	216.0 ± 12.6	215.8 ± 11.2

Values are expressed as mean ± standard error of the mean. WT, wild type; PKD, polycystic kidney disease; C, control; S, sucrose; T, trehalose. * *p* < 0.05 versus the wild-type control group.

## Supplementary Materials

The following are available online at http://www.mdpi.com/2072-6643/11/1/42/s1, Figure S1: No effect of trehalose on the renal expression of autophagy markers in wild-type mice. Figure S2: Immunofluorescence staining for p62 protein in cystic epithelial cells, Table S1: Taqman probe ID and primer sequences.
