# Supplementary material for: Effect of Trehalose Supplementation on Autophagy and Cystogenesis in a Mouse Model of Polycystic Kidney Disease
Source: Nutrients. 2018 Dec 25;11(1):42. doi: 10.3390/nu11010042 (PMC6356442; doi:10.3390/nu11010042)
Supplement: Supplementary file 1 [file nutrients-11-00042-s001.pdf]

Supplementary Table 1. Taqman probe ID and primer sequences

Gene	*Taqman probe or primer sequences (5' to 3')*
Ccl2 (Mcp1)	F: GCTTCTTTGGGACACCTGCTGC<br>R: TTAACGCCCCACTCACCTGCTG
Col1a2	F: AAGGGGTCTTCCTGGTGAAT<br>R: GGGGTACCACGTTCTCCTC
Fn1	F: TGTGACCAGCAACACGGTG<br>R: ACAACAGGAGAGTAGGGCGC
Hmox1(HO1)	F:ACATCGACAGCCCCACCAAGTTCAA<br>R:CTGACGAAGTGACGCCATCTGTGAG
Mmp2	F: TCTGGGCAGTCTTGAACATTT<br>R: AGAGTCAGGTGATGGATGTCG
Tgfb1	F: TGCGTCTGCTGAGGCTCAA<br>R: TTGCTGAGGTATCGCCAGGA
Tnf	F: GCCAGCCGATGGGTTGTA<br>R: GGCAGCCTTGTCCCTTGA
Atg5	Mm01187303_m1
Atg12	Mm00503201_m1
Becn1	Mm01265461_m1
Emr1 (F4/80)	Mm00802529_m1
Hk1	Mm00439344_m1
Hk2	Mm00443385_m1
Il1b	Mm00434228_m1
Il6	Mm01210733_m1
Nos2(iNOS)	Mm00435175_ml
P2rx7	Mm00440578_m1
Rn18S	Mm03928990_g1
Slc2a1	Mm00441480_m1
Slc16a3	Mm00446102_m1
Sqstm1/p62	Mm00448091_m1
Ulk1	Mm00437238_m1

Supplementary Figure 1.

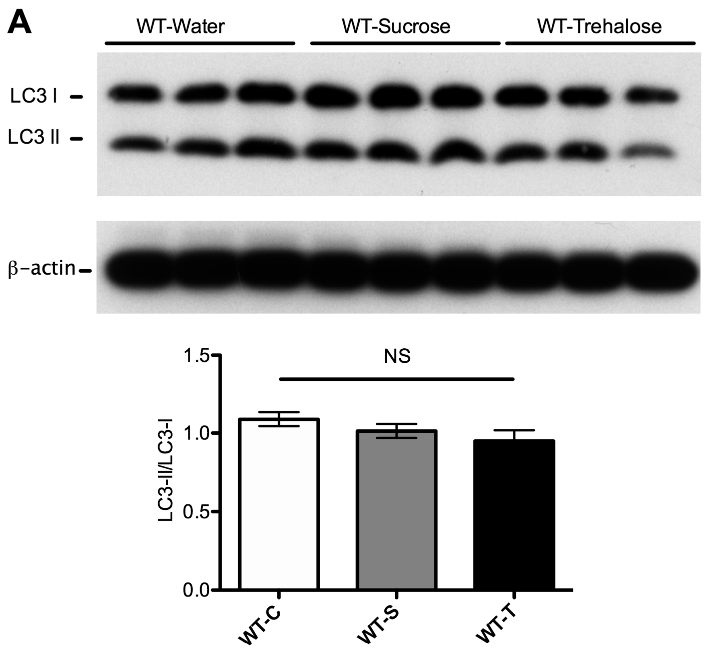

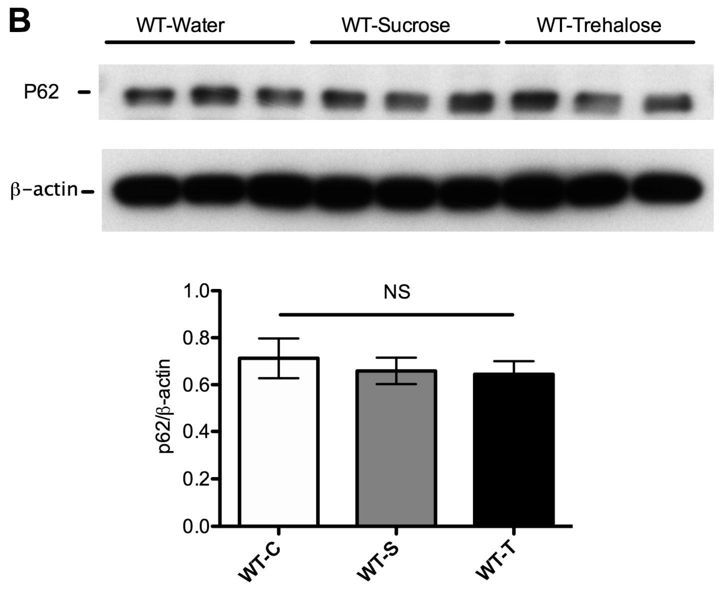

No effect of trehalose on the renal expression of autophagy markers in wild-type mice. The kidney lysates of 3-month-old wild-type mice were immunoblotted for (A) LC3, (B) p62, and β-actin. Graphs represent mean ± standard error of the mean (n = 3 per group). WT, wild-type; C, control; S, sucrose; T, trehalose.

Supplementary Figure 2.

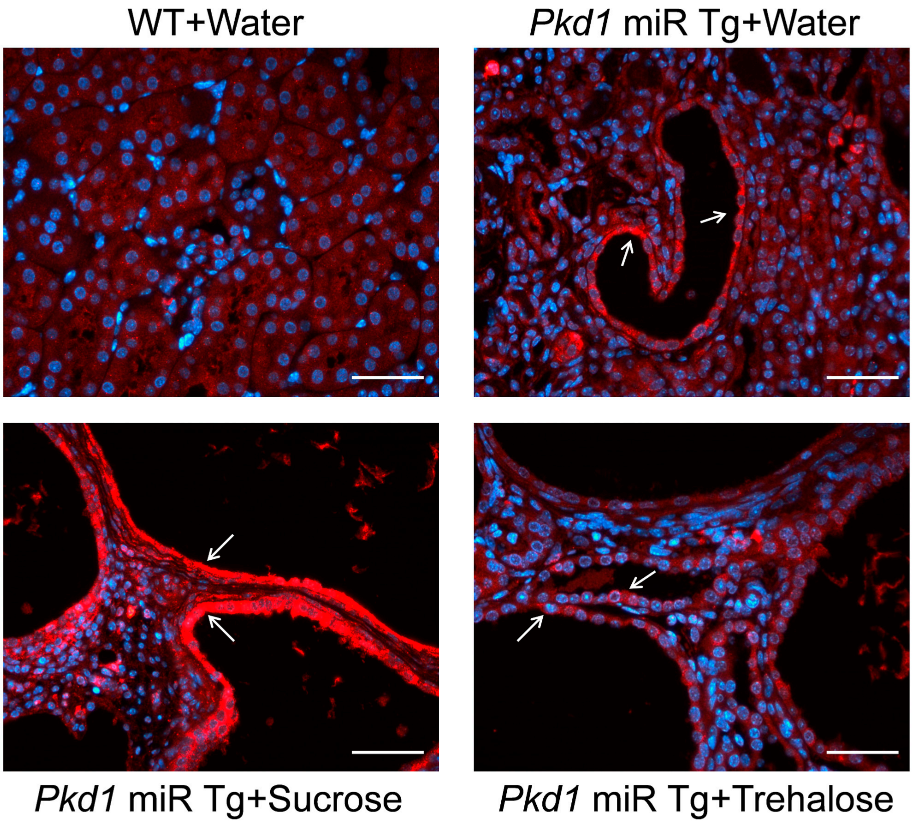

Immunofluorescence staining for p62 protein in cystic epithelial cells. Note p62 staining (shown in red, arrows) of the cystic epithelial cells in *Pkd1* miR Tg mice, indicating reduced autophagic degradation. The nuclei are counterstained with DAPI (blue). No significant differences in the fluorescent p62 signal were found among the different treatment groups. Representative merged images are shown (n=3 per group). Scale bar, 50 μm. WT, wild-type; PKD, polycystic kidney disease. Original magnification, 400×.
